# Which antiarrhythmic drug to choose after electrical cardioversion: A study on non-valvular atrial fibrillation patients

**DOI:** 10.1371/journal.pone.0197352

**Published:** 2018-05-22

**Authors:** Hye Bin Gwag, Kwang Jin Chun, Jin Kyung Hwang, Seung-Jung Park, June Soo Kim, Kyoung-Min Park, Young Keun On

**Affiliations:** 1 Division of Cardiology, Department of Internal Medicine, Heart Vascular Stroke Institute, Samsung Medical Center, Sungkyunkwan University School of Medicine, Seoul, Republic of Korea; 2 Division of Cardiology, Department of Medicine, Kangnam Sacred Heart Hospital, Hallym University Medical Center, Seoul, Korea; University of Messina, ITALY

## Abstract

The relative efficacy of antiarrhythmic drugs (AADs) after electrical cardioversion are not well established. This study aimed to investigate the efficacies of different AADs for maintaining sinus rhythm (SR) after electrical cardioversion for atrial fibrillation (AF). We selected patients from a retrospective registry including patients admitted for cardioversion between January 2012 and June 2016. The primary outcome was time to AF recurrence during the first year after cardioversion. The secondary outcomes included AF recurrence within 1 month, and first readmission due to heart failure, stroke, or additional non-pharmacological rhythm control. A total of 265 patients were divided into the 4 groups according to AAD type: flecainide (n = 33), propafenone (n = 64), amiodarone (n = 128), and dronedarone (n = 40). During the first year after cardioversion, the AF recurrence-free survival was similar between all AAD groups (69.7% vs. 67.2% vs. 71.9% vs. 80.0%, p = 0.439). About half of all recurrences occurred during the first month. There was no difference in any of the secondary outcomes, although the amiodarone group showed a trend toward more non-pharmacological rhythm control. AAD type was not associated with recurrence in multivariate analysis. In this study, half of all patients received amiodarone after electrical cardioversion. Flecainide, propafenone, amiodarone, and dronedarone showed similar efficacies for maintaining SR after electrical cardioversion. Thus, it might be reasonable to reconsider amiodarone use after cardioversion, since it did not show superior efficacy to the other drugs considered and is associated with potential side effects.

## Introduction

Optimal management of atrial fibrillation (AF) requires careful consideration. In particular, the relative merit of rhythm control versus rate control remains unsolved. Although recent randomized trials showed that rate control was not inferior to rhythm control [1–3], rhythm management is required for patients who have substantial symptoms even with well-controlled ventricular rate. Thus, rhythm control strategies can be still useful for patients with symptomatic AF. Electrical cardioversion is one such rhythm control strategy; however, high recurrence rates after cardioversion have highlighted the need for antiarrhythmic drugs (AADs) [4,5]. AADs are widely used for restoration or maintenance of sinus rhythm (SR). However, AADs have demonstrated only modest efficacy and have limited use because of their side effects, including pro-arrhythmia. Recent guidelines [6,7] recommend specific AAD types according to presence or type of concomitant structural heart disease, but the relative efficacy of each agent has not been well demonstrated. In particular, only a few studies have investigated which agent is most effective for maintaining SR after electrical cardioversion [4,5,8,9]. Therefore, we investigated the efficacies of different AADs for maintaining SR after electrical cardioversion for AF.

## Methods

### Study design and patients

We selected patients from the retrospective electrical cardioversion registry of Samsung Medical Center. Consecutive patients admitted to our center between January 2012 and June 2016 for electrical cardioversion due to symptomatic atrial arrhythmia were included in the registry. All patients underwent transesophageal echocardiographic examination before cardioversion to detect thrombus in the left atrial appendage, and anticoagulation was performed for at least 3 weeks in cases of persistent AF or AF with unknown duration before cardioversion. Pre-treatment with AAD was left to the physician’s discretion. Patients who met any of the following criteria were excluded from the analysis: 1) moderate or severe mitral stenosis; 2) previous history of percutaneous mitral balloon valvuloplasty, mitral valve repair, or other valve surgery; 3) congenital heart disease, except for a small atrial or ventricular septal defect; 4) sinus rhythm restoration before cardioversion; 5) thrombus or sludge in the cardiac chamber; 6) previous rhythm control for AF including cardioversion, radiofrequency ablation, or operation; 7) atrial tachyarrhythmia other than AF; or 8) failed cardioversion. Eligible patients were classified into groups according to prescribed AAD type.

### Data collection and clinical outcomes

Clinical and laboratory data were collected by a trained study coordinator using a standardized case report form and protocol. The Institutional Review Board at Samsung Medical Center approved the study protocol (IRB No. 2017-05-022-002). Decisions regarding AAD type and concurrent beta-blocker or calcium channel blocker use were made by the respective physicians. The dose of AAD was maintained in patients on previous AAD therapy, while AAD-naïve patients were treated with the usual starting dose of twice daily administration of flecainide 50mg, propafenone 300mg, amiodarone 200mg, or dronedarone 400mg until the first visit at the clinic. In cases of recurrence, the dose was usually doubled unless the patient was complaining of any side effects. Anticoagulation was continued for at least 4 weeks after cardioversion, and a choice of anticoagulant type (non-vitamin K oral anticoagulant vs. warfarin) was based upon contraindications of each drug and patients preference. All patients were treated with anticoagulants during the first 4 weeks, and treatment was continued at the discretion of physicians. Follow-up visits were routinely performed at 1, 3, 6, and 12 months after electrical cardioversion, with additional visits in the case of any symptom of AF recurrence. The primary outcome was time to AF recurrence during the first year after electrical cardioversion. The secondary outcomes included AF recurrence within 1 month and first readmission due to heart failure, stroke, or additional non-pharmacological rhythm control (including repeated cardioversion, radiofrequency ablation, or operation). Date of AF recurrence was assessed by electrocardiogram or electrocardiographic monitorings. Left ventricular systolic dysfunction was defined as left ventricular ejection fraction < 50%.

### Statistical analysis

Continuous variables are presented as median and interquartile range or mean ± standard deviation, whereas categorical variables are presented as number and percentage. Continuous variables were compared between groups using the Kruskal-Wallis test. Categorical data were compared between groups using Fisher’s exact test or the Chi-square test, as appropriate. For outcome analysis, event-free survival was estimated by the Kaplan-Meier method and compared with the log-rank test. A Cox proportional hazards model was used to adjust for baseline differences between the groups, and variables with a p value < 0.1 were used for adjustment. All tests were two-sided, and a p value < 0.05 was considered statistically significant. IBM SPSS Statistics software version 23 (IBM Corporation, Armonk, NY, USA) was used for statistical analysis.

## Results

### Baseline clinical characteristics

**Fig 1:**
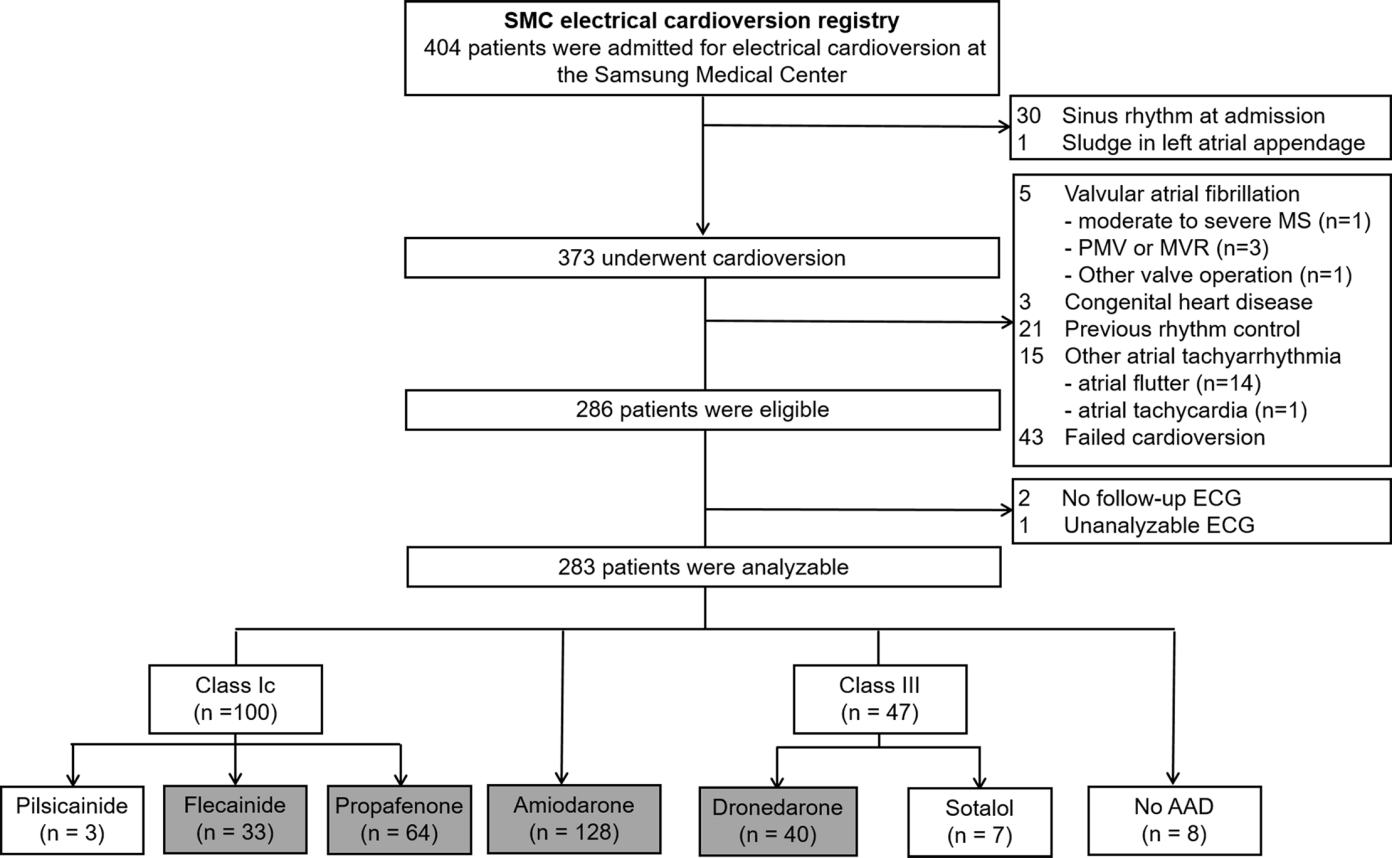
Study population. AAD indicates antiarrhythmic drug; ECG, electrocardiogram; MS, mitral stenosis; MVR, mitral valve replacement; PMV, percutaneous mitral balloon valvuloplasty. AADs in the grey box were included in the final analysis.

A total of 286 patients were eligible for this study. We excluded patients with no follow-up after cardioversion (n = 2) or unanalyzable electrocardiograms (n = 1). Patients who were not on any AAD (n = 8), patients who were on pilsicainide (n = 3), and patients who were on sotalol (n = 7) were also excluded because of their small numbers. Of the final 265 patients, 33 (12.5%) were being treated with flecainide, 64 (24.2%) with propafenone, 128 (48.3%) with amiodarone, and 40 (15.1%) with dronedarone (Fig 1). The patient baseline clinical characteristics are shown in Table 1. The amiodarone group had the highest percentage of males, while the flecainide group had the lowest (92.2% vs. 69.7%, p = 0.007 between the 2 groups). Patients on amiodarone had the highest prevalence of cardiomyopathy and of left ventricular systolic dysfunction; however, there were no statistically significant differences between propafenone and amiodarone group, with the exception of the presence of cardiomyopathy (0% vs. 13.3%, p = 0.001). The amiodarone and dronedarone groups showed higher incidence of coronary artery disease than the propafenone group (p = 0.006 and p = 0.020, respectively). Concurrent use of beta-blockers was more frequent in the flecainide group than in the other groups (p < 0.001). The left atrial volume indexes were similar between the groups, except that the propafenone group had a smaller volume index than the amiodarone group (41.6 [36.1–49.8] vs. 47.5 [40.4–56.2], p = 0.001).

**Table 1:** Patient baseline clinical characteristics.

	Flecainide	Propafenone	Amiodarone	Dronedarone	*P* value
	(n = 33)	(n = 64)	(n = 128)	(n = 40)	
Male	23 (69.7)	53 (82.8)	118 (92.2)	33 (82.5)	0.007
Age (years)	58.0 (51.0–64.0)	59.0 (50.5–65.5)	56.0 (49.0–64.0)	60.0 (56.0–65.5)	0.140
Body mass index (kg/m^2^)	24.7 (22.8–27.0)	25.7 (24.1–27.6)	26.0 (24.0–27.5)	25.3 (23.4–27.6)	0.258
AF duration (months)	6.5 (2.5–49.1)	7.0 (2.4–28.7)	12.4 (3.2–52.3)	13.9 (2.6–60.8)	0.332
Type of AF					>0.999
Paroxysmal	0 (0)	1 (1.6)	1 (0.8)	0 (0)	
Persistent	33 (100)	63 (98.4)	127 (99.2)	40 (100)	
CHA_2_DS_2_VASC score	1.4±1.5	1.3±1.2	1.3±1.5	1.6±1.3	0.347
CHADS_2_ score	0.8±0.9	0.8±0.8	0.8±1.0	1.0±0.9	0.749
Heart failure	1 (3.0)	2 (3.1)	10 (7.8)	1 (2.5)	0.539
Cardiomyopathy	1 (3.0)	0 (0)	17 (13.3)	2 (5.0)	0.021
Ischemic	1 (3.0)	0 (0)	5 (3.9)	1 (2.5)	
Non-ischemic	0 (0)	0 (0)	12 (9.4)	1 (2.5)	
LV systolic dysfunction*	1 (3.3)	5 (7.8)	16 (12.7)	3 (7.5)	0.460
Hypertension	17 (51.5)	29 (45.3)	54 (42.2)	23 (57.5)	0.357
Diabetes	2 (6.1)	9 (14.1)	20 (15.6)	8 (20.0)	0.398
Hyperlipidemia	8 (24.2)	9 (14.1)	19 (14.8)	7 (17.5)	0.586
COPD	0 (0)	0 (0)	3 (2.3)	3 (7.5)	0.102
Current smoking	2 (6.1)	7 (10.9)	15 (11.7)	7 (17.5)	0.538
Current drinking	15 (45.5)	28 (43.8)	66 (51.6)	18 (45.0)	0.724
Previous history of					
Ischemic stroke	3 (9.1)	2 (3.1)	9 (7.0)	2 (5.0)	0.582
Coronary artery disease	2 (6.1)	0 (0)	14 (10.9)	4 (10.0)	0.015
Concurrent medication					
Beta-blocker	22 (66.7)	18 (28.1)	24 (18.8)	6 (15.0)	<0.001
Calcium channel blocker	5 (15.2)	9 (14.1)	18 (14.1)	7 (17.5)	0.962
RASB	6 (18.2)	18 (28.1)	40 (31.3)	14 (35.0)	0.412
Digoxin	2 (6.1)	1 (1.6)	1 (0.8)	0 (0)	0.152
Statin	10 (30.3)	9 (14.1)	24 (18.8)	9 (22.5)	0.270
Echocardiographic measurements*					
LVEF (%)	59.5 (57.0–62.0)	60.0 (56.0–63.0)	59.0 (55.0–64.0)	60.0 (56.0–62.5)	0.913
LA size (mm)	44.0 (40.0–50.0)	42.5 (40.0–47.0)	46.0 (43.0–50.0)	47.0 (41.0–51.0)	0.005
LA volume index (ml/m^2^)	48.3 (37.0–56.1)	41.6 (36.1–49.8)	47.5 (40.4–56.2)	46.3 (37.5–55.4)	0.017

Values are presented as median (interquartile range), mean±SD, or number (percentage).

AAD = antiarrhythmic drug; AF = atrial fibrillation; COPD = chronic obstructive pulmonary disease; LA = left atrial; LVEF = left ventricular ejection fraction; PAOD = peripheral arterial occlusive disease; RASB = renin-angiotensin receptor blocker.

*Echocardiographic measurements were not available for 3 patients in the flecainide group and 2 patients in the amiodarone group.

### Clinical outcomes

**Fig 2:**
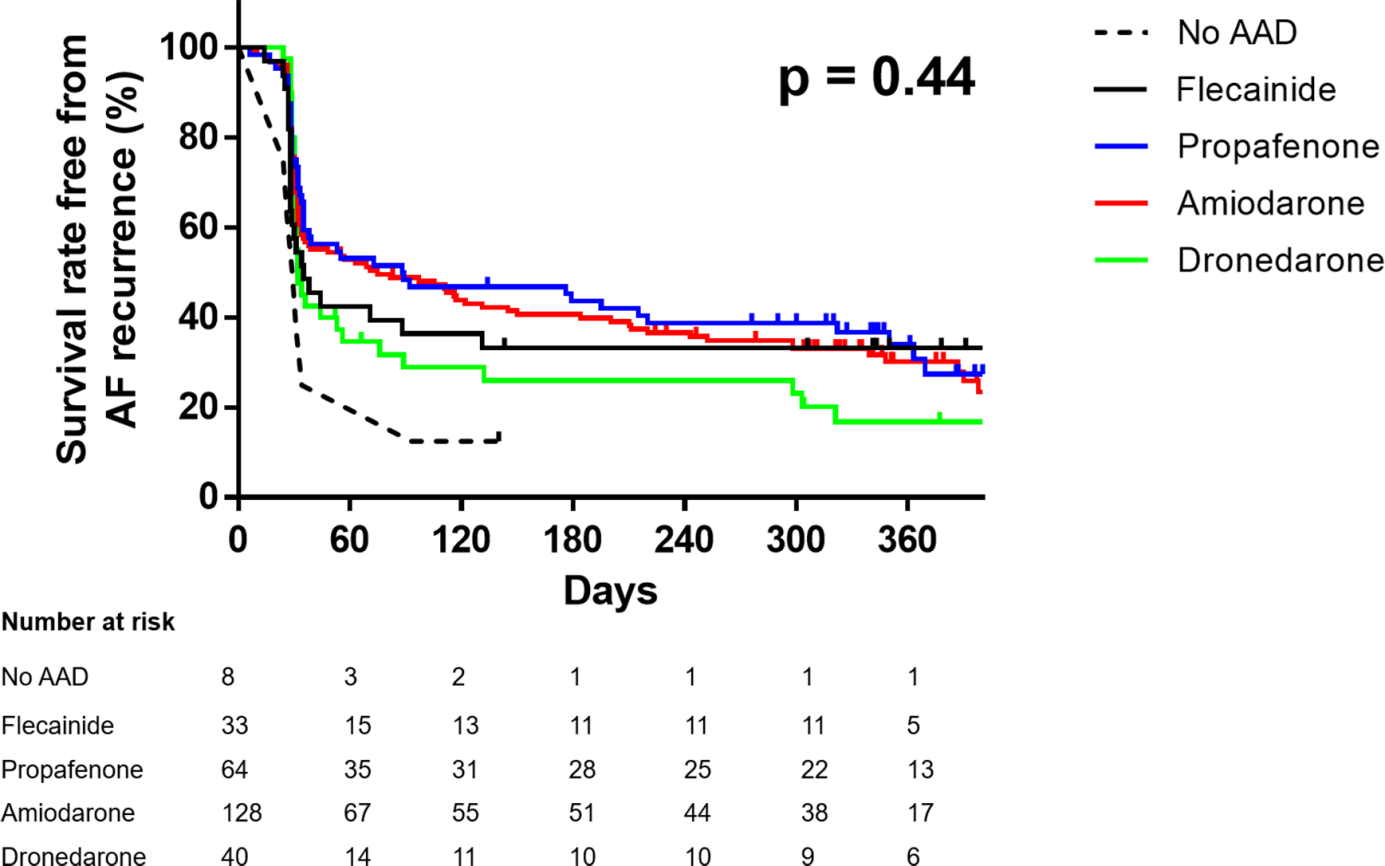
Kaplan-Meier curve for recurrence of atrial fibrillation during the first year after electrical cardioversion according to antiarrhythmic drug type. AF indicates atrial fibrillation; AAD, antiarrhythmic drug. P value as calculated by the log-rank test between the 4 AAD groups. ‘No AAD group’ is shown for reference only.

**Fig 3:**
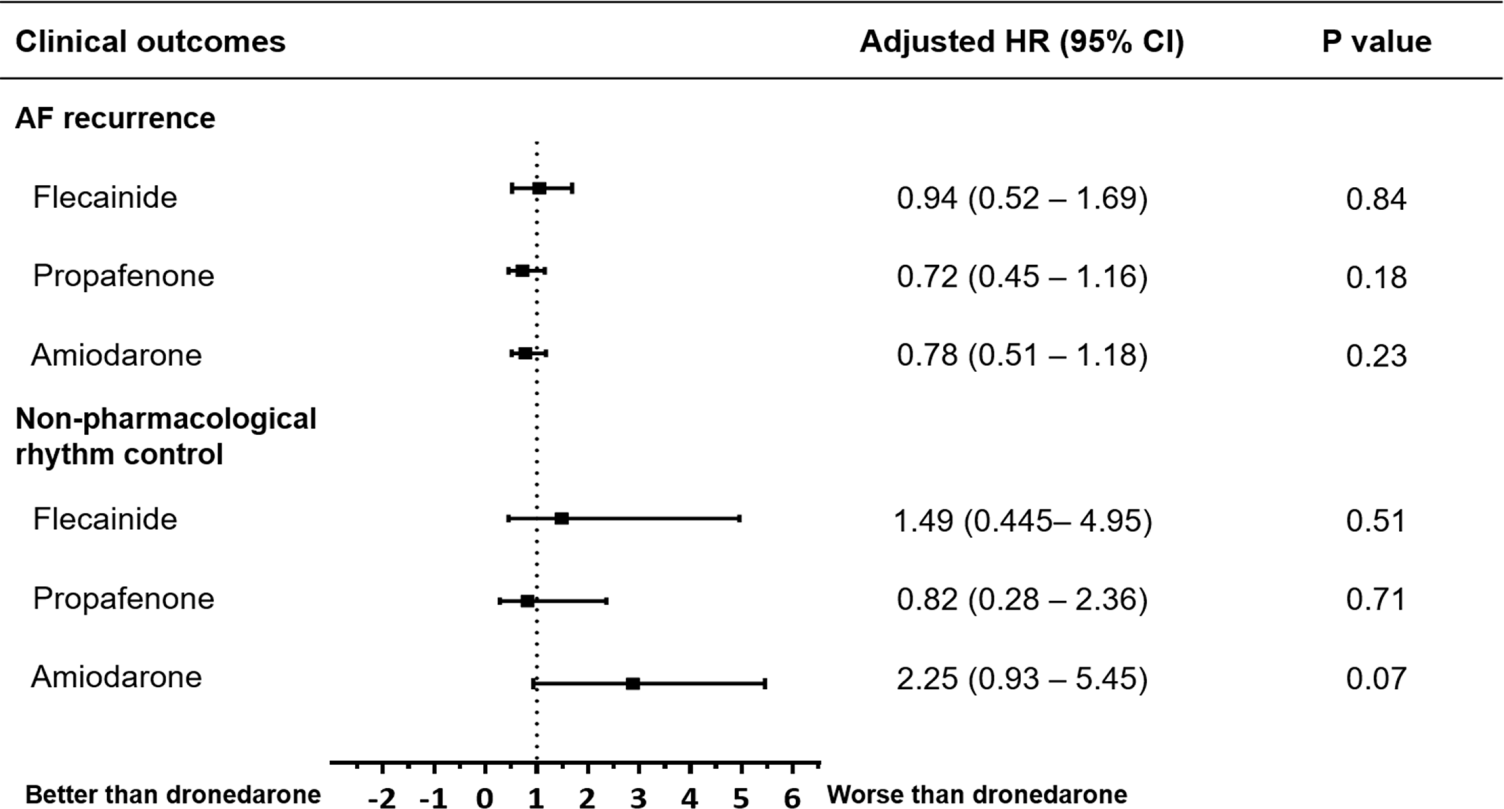
Cox proportional hazards model for atrial fibrillation recurrence and additional rhythm control. The dronedarone group was used as a reference group. Adjusted covariates included sex, cardiomyopathy, chronic obstructive lung disease, coronary artery disease, beta-blocker use, and left atrial volume index. AF indicates atrial fibrillation; CI, confidence interval; HR, hazard ratio.

During the first year after cardioversion, AF recurred in 189 patients (71.3% of the total population), with the highest recurrence rate (80.0%) in patients treated with dronedarone (Fig 2 and Table 2). Log-rank tests between the AAD groups did not reveal any statistically significant differences, although the propafenone and amiodarone groups tended to have lower AF recurrence rates compared to the dronedarone group (p = 0.095 and p = 0.173, respectively, p > 0.5 for the others) (Fig 2). The clinical outcomes are summarized in Table 2 and Fig 3. After adjusting for baseline differences and using dronedarone as a reference, Cox regression analysis did not reveal any association between AAD group and AF recurrence. Only the amiodarone group showed a tendency to be associated with more non-pharmacological rhythm control (hazard ratio 2.25, 95% confidential interval 0.93–5.45, p = 0.074). The rate of anticoagulation therapy at 1 year after cardioversion was lower in the amiodarone group than the propafenone group (75.8% vs. 89.1%, p = 0.034), while there were no differences between the other groups (S1 Table).

**Table 2:** Clinical outcomes according to antiarrhythmic drug group.

	Flecainide	Propafenone	Amiodarone	Dronedarone	†p value
	(n = 33)	(n = 64)	(n = 128)	(n = 40)	
AF recurrence during 1 year	23 (69.7)	43 (67.2)	91 (71.9)	32 (80.0)	0.563
AF recurrence within 1 month	19 (57.6)	29 (46.0)*	60 (51.3)*	26 (66.7)*	0.208
Admission due to heart failure	1 (3.0)	1 (1.6)	1 (0.8)	1 (2.5)	0.497
Stroke	0 (0)	0 (0)	2 (1.6)	1 (2.5)	0.612
Non-pharmacological rhythm control	7 (21.2)	9 (14.1)	35 (27.3)	6 (15.0)	0.127
Repeated cardioversion	2 (6.1)	0 (0)	4 (3.1)	1 (2.5)	
RF ablation	1 (3.0)	7 (10.9)	12 (9.4)	2 (5.0)	
Operation	4 (12.1)	2 (3.1)	19 (14.8)	3 (7.5)	

Values are presented as n (%).

†P value refers to the difference among the groups as assessed by the chi-square test.

AAD = antiarrhythmic drug; AF = atrial fibrillation; CI = confidence interval; HR = hazard ratio; RF = radiofrequency.

*One-month ECG was not available for 1 patient in the propafenone, 11 patients for the amiodarone group, and 1 patient for the dronedarone group.

## Discussion

In the present study, we investigated the relative efficacies of AADs for maintaining SR after electrical cardioversion. The major findings of this study were: (1) the recurrence rate was 71.4% during the first year after electrical cardioversion, with approximately half of all recurrences occur within the first month; and (2) no AAD showed superior efficacy compared to the others, although amiodarone did show a tendency for greater additional rhythm control.

Setting aside the debate about whether rhythm control or rate control is a better strategy, cardioversion is still a useful option for patients with symptomatic AF. However, SR maintenance after cardioversion is challenging, with recurrence rates ranging from 63–84% in the first year [4,5]. While several types of AADs are available, their efficacies are modest at best, and they are associated with undesirable side effects. Therefore, safety has been proposed to be more important than efficacy when choosing the type of AAD [6,7]. Nevertheless, data regarding the relative efficacy of AADs would provide additional information for guiding AAD choice. Although some studies have focused on this subject, none was a prospective randomized trial. Furthermore, direct comparison between all AAD types could not be performed, since previous randomized trials conducted head-to-head comparison between two AAD groups or between an AAD group and a placebo group [9–12]. One review and a recent prospective cohort study presented the relative efficacies of different AADs for prevention of recurrent AF after electrical cardioversion [4,8]. The review reported amiodarone as the most effective agent (Peto odds ratio 0.19 [0.14–0.27]). Similarly, the cohort study concluded that amiodarone seemed to be superior to the other AADs, even in patients without structural heart disease. In contrast to these studies, all AADs included in the present study showed similar efficacy for maintaining SR. The difference between our results and theirs might resulted be due to differences in study design, study population, or specific study limitations. The review was based on previously published data lacking randomized trials and the author’s personal experience. In the other study, only patients without structural heart disease were included, and analyses did not adjust for baseline differences in each AAD group, even though AAD use was not randomized. The recurrence rates were only compared for each time period; moreover, amiodarone only had a trend of better efficacy compared to the other class 1c agents, without statistically significant difference (p = 0.09).

Since amiodarone use as first-line treatment has been limited due to possible extracardiac side effects, it has been recommended to reserve this drug for specific situations such as concomitant heart failure. However, similar to previous studies, amiodarone was the most frequently prescribed drug in our study (48.1%). In the previous prospective registry study, 55% of all patients were treated with amiodarone, even though the study population consisted of patients without structural heart disease. The reason why amiodarone was the most commonly used agent in these studies is unclear. We postulate that amiodarone was the second-line treatment in these studies, because it is likely that electrical cardioversion was attempted in patients who were refractory to their previous first-line treatment. In our study, the rate of de novo AAD use was significantly lower in the amiodarone group than in all other groups (22.7% in the amiodarone, 90.9% in the flecainide group, 37.5% in the propafenone group, and 45% in the dronedarone group, p < 0.001). Therefore, the antiarrhythmic efficacy of amiodarone could have been underestimated due to potential bias by including more patients refractory to first-line therapy in the amiodarone group. Of note, a recent large retrospective study comparing the efficacies of different first-line AADs in AF patients demonstrated that amiodarone showed the best efficacy for prevention of AF recurrence [13]. This finding might be explained by the same rationale as described above. For this reason, we performed a subgroup analysis targeting only patients on de novo AADs. The prevalence of heart failure, left ventricular systolic dysfunction, cardiomyopathy, and concomitant beta-blocker use was higher in the amiodarone group than in all other AAD groups. There was no difference in AF recurrence-free survival rate between the groups, and AAD type was not associated with AF recurrence, even after multivariate adjustment (S1 Fig). However, the multivariate analysis was underpowered due to the small number of patients on de novo therapy (30 patients with flecainide, 24 with propafenone, 29 with amiodarone, and 18 with dronedarone). Because amiodarone is preferred for patients with structural heart disease or heart failure, there is a possibility of underestimated efficacy of amiodarone. We also cannot rule out other unmeasured confounding factors influencing both prescription of amiodarone and recurrence of AF.

Another interesting observation in our study was the high percentage of male patients. We postulate that this was partly because of a higher incidence of AF in men. Males have a higher incidence of AF in all age groups, while the absolute number of patients seems equal in the elderly population because the incidence of AF and the proportion of females increase with age. Because most patients in our study were in their 50s and 60s, it is likely that more men were included than women. A similar trend was observed in previous studies investigating Korean AF patients undergoing cardioversion (74.0% ~ 80.6% males in total population) [9,14,15].

The concept of upstream therapy was introduced to reduce or prevent atrial remodeling, which perpetuates AF. Several agents such as renin-angiotensin receptor blockers, statins, or polyunsaturated fatty acids have been studied as part of this therapy [16–18]. As with the previous studies, the present study does not rule out the possibility that these non-AADs have antiarrhythmic effects, even though these agents have not yet shown convincing benefits. Additionally, the results could have been confounded by the effects of other factors, like the antiarrhythmic effects of beta-blockers and calcium channel blockers [5,19,20], management of concurrent cardiovascular conditions, and/or lifestyle factors (exercise intensity, weight reduction, smoking, and alcohol intake) [21,22].

Considering all evidence obtained to date, it seems reasonable to reserve amiodarone only for special situations, since it was not shown to be superior to the other AADs and is associated with potential side effects. However, the lack of data and the limitations of previous studies comparing AADs emphasize the need for cautious interpretation of the previous results and for further randomized trials. In particular, we anticipate that future studies would be of great use to clinical practice by investigating optimal duration of AAD therapy and AAD choice following treatment failure.

There were several limitations in this study. First, this was a retrospective single-center study. Not all patients could be followed-up according to a standardized protocol, and there is a chance that symptomatic patients could have visited the clinic more frequently. There were also differences in baseline characteristics between the 4 groups. Even though we conducted multivariate analysis to adjust for these differences, the possibility of confounding effects remains. Second, patients on pilsicanide and sotalol were excluded from the study due to their small numbers. Third, as discussed above, we could not validate the antiarrhythmic effect of drugs other than AADs. Fourth, we could not assess other clinical outcomes including quality of life. Lastly, we investigated clinical outcomes during a follow-up duration of one year. While this time period might seem short, the outcomes would probably not have been much different with longer follow-up times, considering that most of the recurrences occurred during the first 6 months and reached a plateau thereafter.

In this retrospective study, flecainide, propafenone, amiodarone, and dronedarone showed similar efficacies for maintaining SR after electrical cardioversion. Since amiodarone did not show superior efficacy to any other AAD and is associated with potential side effects, it might be reasonable to reserve amiodarone for special situations. However, our results need to be interpreted with caution because of the retrospective observational design of the study.

## Supporting information

S1 Table Anticoagulation therapy after electrical cardioversion. (DOCX)

S1 Fig Kaplan-Meier curve for recurrence of atrial fibrillation during the first year after electrical cardioversion according to type of antiarrhythmic drugs (AADs) in patients on de novo AADs. AF indicates atrial fibrillation; AAD, antiarrhythmic drug. P value as calculated by the log-rank test between the 4 AAD groups. ‘No AAD group’ is shown for reference only.
(TIF)

S1 Dataset Minimal relevant dataset of this study. (XLSX)
