# Supplementary material for: Which antiarrhythmic drug to choose after electrical cardioversion: A study on non-valvular atrial fibrillation patients
Source: PLoS One. 2018 May 22;13(5):e0197352. doi: 10.1371/journal.pone.0197352 (PMC5963785; doi:10.1371/journal.pone.0197352)
Supplement: S1 Table — (DOCX) [file pone.0197352.s001.docx]

**S1 Table.** Anticoagulation therapy after electrical cardioversion

|  | Flecainide | Propafenone | Amiodarone | Dronedarone | †p value |
| --- | --- | --- | --- | --- | --- |
|  | (n=33) | (n=64) | (n=128) | (n=40) |  |
| Continuation therapy at 1 year | 30 (90.9) | 57 (89.1) | 97 (75.8) | 35 (87.5) | 0.039 |
| Type of anticoagulant at discharge |  |  |  |  | < 0.001 |
| Warfarin | 4 (12.1) | 32 (50.0) | 42 (32.8) | 22 (55.0) |  |
| Non-vitamin K antagonist | 29 (87.9) | 32 (50.0) | 86 (67.2) | 18 (45.0) |  |

Values are presented as n (%). †P value refers to the difference among the groups as assessed by the chi-square test.
