# Supplementary figures and images for: Which antiarrhythmic drug to choose after electrical cardioversion: A study on non-valvular atrial fibrillation patients
Source: PLoS One. 2018 May 22;13(5):e0197352. doi: 10.1371/journal.pone.0197352 (PMC5963785; doi:10.1371/journal.pone.0197352)

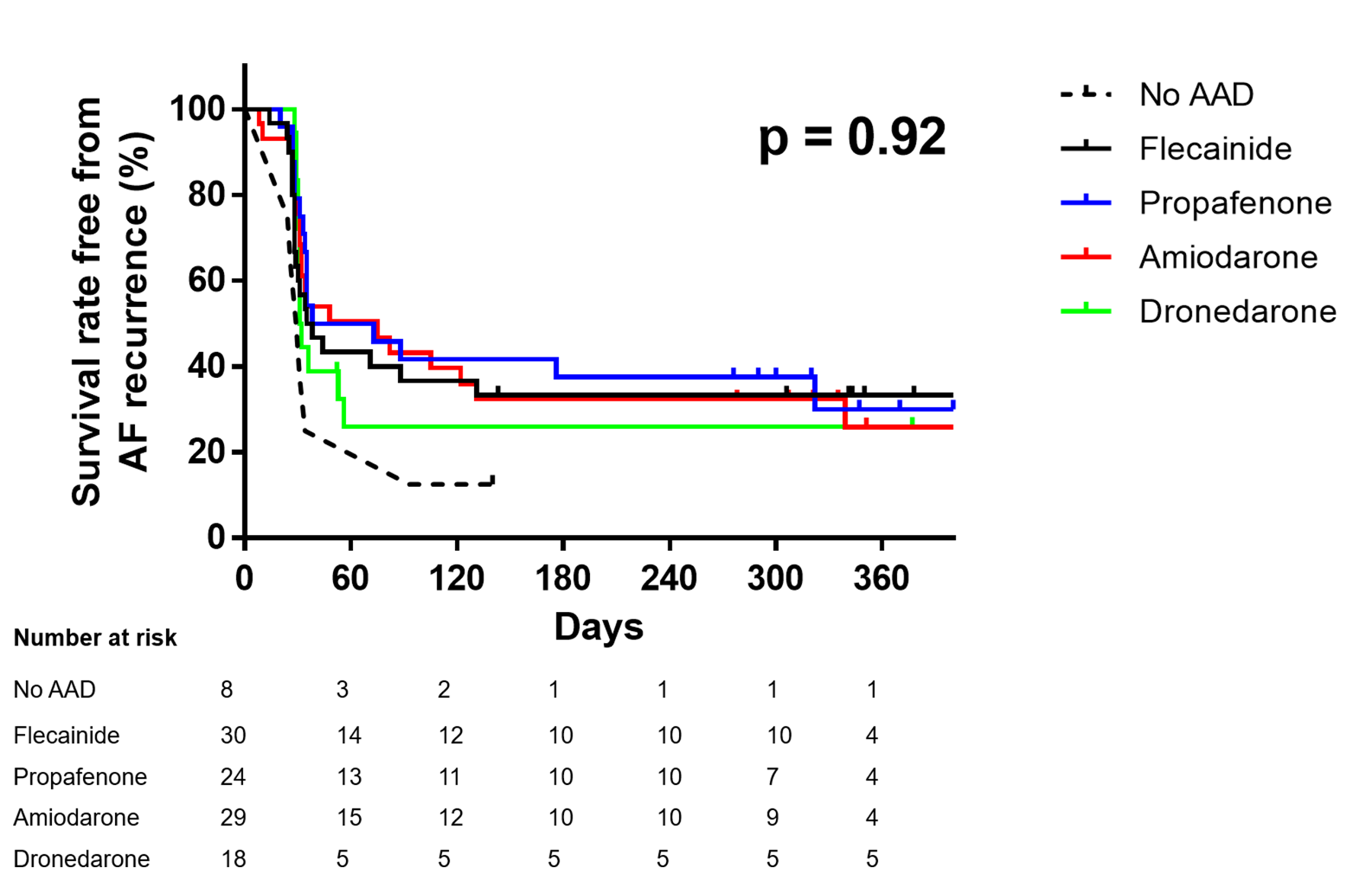

Supplement: S1 Fig — AF indicates atrial fibrillation; AAD, antiarrhythmic drug. P value as calculated by the log-rank test between the 4 AAD groups. ‘No AAD group’ is shown for reference only. (TIF) [file pone.0197352.s002.tif]
